# Meditation Mobile App Developed for Patients With and Survivors of Cancer: Feasibility Randomized Controlled Trial

**DOI:** 10.2196/39228

**Published:** 2022-11-23

**Authors:** Jennifer Huberty, Nishat Bhuiyan, Megan Puzia, Lynda Joeman, Linda Larkey, Ruben Mesa

**Affiliations:** 1 Mays Cancer Center University of Texas Health San Antonio MD Anderson Phoenix, TX United States; 2 College of Health Solutions Arizona State University Phoenix, AZ United States; 3 Behavioral Research and Analytics, LLC Salt Lake City, UT United States; 4 Lynda Joeman Research Consultancy Tonbridge United Kingdom; 5 Edson College of Nursing and Health Innovation Arizona State University Phoenix, AZ United States; 6 Mays Cancer Center University of Texas Health San Antonio MD Anderson San Antonio, TX United States

**Keywords:** cancer, mobile health, mHealth, meditation, feasibility, mobile phone

## Abstract

**Background:**

To address the unmet need for a commercial cancer-specific meditation app, we leveraged a long-standing partnership with a consumer-based app (ie, *Calm*) to develop the first commercial meditation app prototype adapted specifically for the needs of patients with cancer. Input was obtained at both the individual user and clinic levels (ie, patients with and survivors of cancer and health care providers).

**Objective:**

This study aimed to determine the feasibility of a cancer-specific meditation app prototype.

**Methods:**

Patients with and survivors of cancer who were recruited and enrolled in the feasibility randomized controlled trial were asked to use the prototype app daily (≥70 minutes per week) for 4 weeks. Participants completed web-based weekly questionnaires and a final poststudy questionnaire and were asked to participate in an optional web-based poststudy interview. The questionnaires and interviews covered the following feasibility categories: acceptability, demand, practicality, and adaptation.

**Results:**

A total of 36 patients with and survivors of cancer completed the baseline questionnaire, 18 completed the final questionnaire, and 6 completed the optional interviews. Weekly and poststudy questionnaires indicated high overall enjoyment, ease of use, and satisfaction with the app content, aesthetics, and graphics. The objective use data indicated that the average total app use rate was 73.39 (SD 7.12) minutes per week. Interviews (N=6) revealed positive and mixed responses to the app prototype and informative differences related to preferences for narrators, emotional content, and meditation teaching but an overall appreciation for the variety of options.

**Conclusions:**

The most likely candidates for moving from cancer-specific meditation apps to dissemination are through partnering with the industry, in which name recognition and market distribution are already established (even showing a base of users from the targeted population with cancer). This study established the feasibility of a cancer-specific mobile meditation app prototype for patients with and survivors of cancer, using a commercially available app. The quantitative and qualitative data demonstrated the acceptability, demand, practicality, and adaptation of the prototype. Improvements suggested by the participants will be considered in the final app design before testing the efficacy of the app in a future study.

**Trial Registration:**

Clinicaltrials.gov NCT05459168; https://clinicaltrials.gov/ct2/show/record/NCT05459168

## Introduction

### Background

The need for effective nonpharmacologic strategies, such as meditation, to manage the debilitating and costly chronic symptom burden faced by patients with and survivors of cancer has been well established [1-3]. Furthermore, there is a clear need to translate in-person delivered meditation programs into more accessible and maintainable formats for patients with and survivors of cancer owing to the costs, limited sustainability, and patient-reported barriers (eg, distance, scheduling, and symptom burden) associated with in-person programs [4]. One promising method for delivering meditation programs to patients with and survivors of cancer is the use of mobile health apps. Research has clearly demonstrated the short-term benefits of mobile apps and web-based meditation programs [5,6]. Reports indicate that as many as 97% of patients with and survivors of cancer have access to smartphones and are willing to use app-based meditation [5,6].

Evidence suggests that more general meditation apps do not sufficiently meet the unique physical, emotional, and social needs of patients with and survivors of cancer [7-9]. Populations with cancer may have specific fears (fear of cancer progression, recurrence, etc) and stressors that can arise during meditation, physical limitations to seated meditation, symptom-monitoring needs, and survivor-related social support needs specific to meditation that could be addressed within a cancer-specific meditation app [7-9]. Furthermore, there are significant limitations associated with existing commercial meditation apps, including not being specifically tailored toward populations with cancer and not including inputs from patients with and survivors of cancer before development. To date, there are no commercially available evidence-based apps for patients with cancer that are specifically devoted to meditation. A cancer-specific meditation app is necessary to ensure clinical acceptability, effectiveness, and safety for this population. The most likely candidates for moving from cancer-specific meditation apps to dissemination are through partnering with the industry, in which name recognition and market distribution are already established (even showing a base of users from the targeted population with cancer).

The primary author developed a partnership with a consumer-based app (ie, *Calm*) to conduct investigator-initiated research with broad reach and potential for sustainability in a variety of populations. We leveraged the long-standing partnership with *Calm* to develop the first commercial meditation app prototype adapted specifically for the needs of patients with cancer, titled *Calm for Cancer*. Details regarding the development of this app have been reported elsewhere [10]. Briefly, the development of the *Calm for Cancer* app was guided by the Integrate, Design, Assess, and Share framework [11] and input from an advisory committee of patients with and survivors of cancer. We worked with not-for-profit partners to ensure that there was representation of the advisory committee (end users at both the individual and clinic levels) who contributed to the prototype design and development. Specifically, the committee included patients with and survivors of cancer, health care providers, and subscribers of the parent app, *Calm*, who participated in surveys, daily journals, and focus groups. Insights from the advisory committee were integrated into the development process to build the *Calm for Cancer* prototype’s content and features [10].

### Objectives

The purpose of this paper was to report the findings of a 4-week feasibility randomized controlled trial (RCT) of the *Calm for Cancer* app among patients with and survivors of cancer. Using the feasibility model by Bowen et al [12] as a guiding framework, we aimed to determine the acceptability, demand, practicality, and adaptation of the *Calm for Cancer* app using questionnaires and objective use data. The secondary aim was to explore the associations between app use and satisfaction outcomes. Finally, we aimed to gain further insight into the overall experiences of the patients with and survivors of cancer with the meditation app prototype via qualitative in-depth interviews. The findings will help develop the next phase of the prototype to be tested for its efficacy.

## Methods

### Ethics Approval

All study materials and procedures were approved by the institutional review board of Arizona State University (protocol ID STUDY00011444).

### Overview

Study recruitment took place between October 7, 2021, and December 10, 2021, with participants enrolled in the study on a rolling basis. Patients with and survivors of cancer were recruited nationwide, using internet-based strategies, including social media (ie, Facebook, Twitter, and Instagram), various cancer groups’ and organizations’ listservs and websites, closed social media groups, and by contacting patients with cancer who were ineligible for prior web-based studies and who had consented to being contacted about future research opportunities. The research team provided flyers to recruitment contacts, which included a description of the study and a link to the web-based eligibility screening survey. In an effort to recruit racially and ethnically diverse patients, recruitment materials included the statement, “We are looking for patients with and survivors of cancer who represent diverse racial and ethnic groups, gender, and cancer types and those who have never meditated.” Partner cancer groups, organizations, and closed social media groups were asked to focus on diverse patients when sharing recruitment materials. Patients and survivors who were interested in participating completed the eligibility survey via REDCap (Research Electronic Data Capture; Vanderbilt University) [13]. Potential participants were eligible for the study if they (1) had a cancer diagnosis within the past 2 years, (2) were aged ≥18 years, (3) owned an iPhone or iPad with iOS 9.0 or later, (4) were willing to download a mobile app, (5) were able to read and understand English, and (6) were not currently engaged in a regular meditative movement practice (eg, yoga, tai chi, or qi gong with substantial meditation) for ≥60 minutes per week in the past 6 months. The eligibility survey took approximately 5 to 10 minutes to complete. Participants who did not meet the eligibility criteria were sent an email notification regarding their eligibility status and the reasons for ineligibility.

Eligible participants were emailed a link to an informed consent page via REDCap with details about the requirements of the study as well as potential risks and benefits. Those who agreed to participate were instructed to type their electronic signatures, which constituted their consent to participate in the study. After receiving the signed consent form, participants were sent an email containing (1) an overview of the study procedures, (2) a link to the baseline demographic questionnaire, (3) instructions for downloading and using the *Calm for Cancer* app prototype, and (4) instructions and an invitation link to join a private *Calm for Cancer* Facebook support group (detailed in further sections). In addition to the baseline demographic questionnaire, participants were asked to complete 4 weekly satisfaction questionnaires on the web via REDCap and a final overall study satisfaction questionnaire via REDCap. The weekly satisfaction questionnaires took approximately 5 minutes to complete, and the final overall study satisfaction questionnaire took approximately 15 to 20 minutes to complete. Finally, after completing the 4-week feasibility RCT, participants were given the option to participate in an in-depth interview to discuss their overall experience of using the *Calm for Cancer* app. The interviews were conducted by a member of the research team via Zoom teleconferencing software (Zoom Video Communications). The interview questions are presented in Multimedia Appendix 1. The participants had the option of not participating in the interview if they did not want their interviews to be recorded. Each interview took approximately 30 minutes to complete. Participants received US $5 for submitting each weekly satisfaction questionnaire, US $20 for completing the final poststudy satisfaction questionnaire, and US $20 for completing the virtual poststudy interview (up to US $60 for completing all study questionnaires and the interview).

### The Calm for Cancer Prototype

The *Calm for Cancer* prototype was a stand-alone app available for download in the Apple store and only available to research participants. Participants were asked to use the *Calm for Cancer* prototype for 4 weeks at any time for at least 10 minutes per day but were encouraged to use it as much as they would like, mimicking how a new paying member would use the original *Calm* app. This dose was chosen because positive effects have been reported even after 10 minutes of daily meditation practice [14-16], which is consistent with our work in patients with hematologic conditions [5,6]. Participants were also encouraged to explore and use the different features of the app, such as the optional prompts to rate the session at the end of the meditation sessions, setting meditation reminders, and tracking their participation within the app.

The *Calm for Cancer* prototype contained a variety of guided meditations and breathing exercises that were developed based on our formative work with patients with and survivors of cancer and health care providers [8]. These include (1) introductory meditations: 7 days of basic meditation that introduced meditation practices to the user; (2) calming the chaos and uncertainty meditations: a series of 23 brief daily meditations that were guided by storytelling; (3) meditations with an oncologist: a series of meditations to help the user understand the science behind meditation and its benefits; (4) meditations for the authentic self: a series of guided meditations for self-love; (5) power of breath: a series of breath work practices to cultivate gratitude, self-kindness and so on; (6) reset your emotions: a series of brief 2- to 3-minute meditation sessions to help reset emotions in times of panic and anxiety (eg, in the doctor’s office); (7) meditations with a survivor of cancer: a series of meditations led by a survivor of cancer; (8) content from the original *Calm* app: various guided meditations and master classes not specific but relevant to patients with and survivors of cancer to help with processing and acceptance, managing emotions, and working with anxiety and fear; (9) sleep stories from the original *Calm* app: narrated fictional tales to help promote sleep; and (10) calming music: an assortment of relaxing music that patients with and survivors of cancer can listen to during treatments or at their own leisure.

The app was built upon a variety of behavior-change strategies known to improve behavior, as well as insights from patients with and survivors of cancer and the advisory committee. First, meditations and other content in the *Calm for Cancer* prototype app were led by yoga instructors and meditation teachers specializing in cancer or who had personal experience with cancer (ie, themselves or immediate family), spiritual teachers, and oncologists. The teachers were male and female and represented a variety of racial minority groups. Tailored interventions have been shown to be more effective in improving outcomes than nontailored interventions. In addition, patients with and survivors of cancer may be more receptive to content when guided by someone with a similar experience and when a sense of relatedness is felt [17,18]. Second, the *Calm for Cancer* prototype included a feature that allowed participants to complete check-ins in which they could log their emotions, self-reflections, gratitude, and sleep. Patients and survivors wanted the ability to monitor how they feel and observe the changes over time. Providing feedback or SMS text messaging based on user performance may also help increase adherence, and users are more likely to use an app with a self-monitoring mechanism [19,20]. Third, because patients with and survivors of cancer want to connect with others to support belonging, and social media-based support groups for patients with and survivors of cancer have increased knowledge and decreased anxiety in app-based interventions, the *Calm for Cancer* prototype also allowed the user to share their meditation participation with others via social media.

### Facebook Group

Because our formative work suggested that patients with and survivors of cancer wanted a place to discuss their meditation participation (but not within the app or with their physician), the research team developed a private *Calm for Cancer* support group on Facebook. The Facebook group served as an informal platform where participants could connect with other study participants to share their experiences with meditation related to their cancer. The Facebook group had a moderator (ie, a member of the research team) and rules about what could and could not be posted (eg, no derogatory comments or profanity). Participants were asked to log on to the page at least once per week *but were not required to adhere to any other criteria for engagement (eg, number of posts) other than the weekly log-in*. Every week, the moderator posted a discussion prompt to encourage participation; the complete list of Facebook discussion prompts is available in Multimedia Appendix 2.

### Measures

#### Demographics

Participants self-reported the following demographic characteristics at baseline: sex, ethnicity, race, annual income, education, employment status, marital status, and cancer diagnosis.

#### Feasibility

Feasibility questionnaires and interviews were based on the feasibility model by Bowen et al [11]. Bowen et al [11] posit that for an intervention to be worthy of testing its efficacy, relevant questions must be addressed within feasibility. The key areas of focus for feasibility according to Bowen et al [11] were used here and included the following: (1) acceptability (ie, satisfaction, perceived appropriateness, and perceived positive and negative effects), (2) demand (ie, use of the app, interest, or intention to use), (3) practicality (ie, how it makes them feel and ease of use), and (4) adaptation (ie, suggestions for modifications to improve the app to better meet the unique needs of patients with and survivors of cancer). Bowen et al [11] suggest that methodologies used to address each area can vary and be creatively combined to fit the setting, community, or population under study. Feasibility outcomes were assessed via 4 weekly questionnaires, a final poststudy questionnaire, and in-depth poststudy interviews. The questionnaires and interviews were developed by a team of research investigators (ie, oncologists and researchers in cancer, mobile apps, and meditation).

#### Use (Demand)

In addition to the questionnaires and interviews, the demand for the app was further assessed through objective participation (ie, use data). Participation (ie, use data) were automatically tracked within the *Calm for Cancer* prototype over 4 weeks and provided to the research team by *Calm*. The objective participation (ie, use) data included the total number of meditation sessions completed within the app, total number of minutes using the app, and specific components used (ie, master class, series, music, sleep story, freeform, and sequential).

### Interviews

The purpose of the qualitative in-depth interviews was to gain further insight into the participants’ overall experience using the *Calm for Cancer* app prototype. Interview questions included, “Tell us about your experience using the meditation app prototype,” “Were there things that were missing from your experience with the app that you would have benefited from?” and “Do you think other patients with and survivors of cancer will have any difficulty accessing or using the meditation app? Describe why or why not.” The complete semistructured interview guide is available in Multimedia Appendix 1.

### Statistical Analysis

Descriptive statistics and frequencies were computed to describe sample baseline characteristics and to summarize weekly self-reported feasibility outcomes, poststudy self-reported feasibility outcomes, and objective use data. As an additional exploratory analysis, linear regression models were used to examine whether app use (ie, total minutes of app use per week and total completed meditation sessions per week) predicted participants’ self-reported overall enjoyment and satisfaction with content, ease of use, aesthetics, and graphics. All statistical analyses were performed using SPSS (version 28.0; IBM Corp), with significance inferred at *P*<.05.

### Qualitative Analysis

The interview transcripts were imported into NVivo 12 qualitative analysis software (QSR International) for coding and analysis. Top-level themes were identified deductively based on the main information requirements of the study and topics covered in the interview guide. Within these, lower-level themes and findings were inductively identified from the interview transcripts.

## Results

### Overview

A total of 120 patients with cancer completed the eligibility screening survey, and 50 (41.7%) met the eligibility criteria and consented to participate in the study. Of these 50 participants, 36 (72%) completed the baseline questionnaire, of these 36 participants, 18 (50% of baseline questionnaire completers) participants completed the final poststudy questionnaire. Reasons for dropout included 17 participants lost to follow-up (ie, participants who did not complete additional surveys and who were unable to be contacted further) and 1 participant who declined further participation. A total of 6 participants completed the optional poststudy interviews. As shown in Table 1, most participants were female, non-Hispanic, White, and had a relatively high socioeconomic status (ie, had completed a Bachelor’s degree or higher, were employed, and had an annual household income of ≥US $100,000). The most common type of cancer was blood cancer (10/36, 28%), followed by breast cancer (9/36, 25%). Approximately half of the participants (18/36, 50%) received their first cancer diagnosis between 1 and 3 years ago.

As shown in Table 2, weekly questionnaire responses indicated enjoyment, ease of use, and satisfaction with the meditation app prototype.

As shown in Table 3, poststudy questionnaire responses also indicated enjoyment, perceived ease of use, satisfaction with the meditation app content, and satisfaction with the meditation app aesthetics and graphics after the study. Similar to the weekly survey responses, most participants (10/11, 91%) enjoyed or very much enjoyed using the meditation app prototype; found the meditation app prototype easy or very easy to use; and were satisfied or very satisfied with the meditation content, aesthetics, and graphics. Most users found that the length of meditation sessions was just right (9/11, 82%) and found that the app was helpful for improving or managing symptoms or difficulties related to cancer (10/11, 91%). All participants (11/11, 100%) who responded indicated that they would recommend the *Calm for Cancer* meditation app prototype to other patients with and survivors of cancer and that they were likely or extremely likely to continue using the app. Participation in the *Calm for Cancer* Facebook group was low among app users; a total of 14 participants accepted the invitation and joined the group, and only 4 participants responded to weekly discussion posts. In the poststudy period, most participants (7/9, 78%) rated their overall satisfaction with the Facebook group as neutral.

On average, enrolled participants (who completed surveys at least at week 1) used the app (ie, total app use, including meditations and sleep content) for 73.39 (SD 7.12) minutes per week and completed 4.92 (SD 1.05) sessions per week. More specifically, participants completed an average of 4.62 (SD 1.29) meditation sessions, 0.19 (SD 0.25) music sessions, and 0.11 (SD 0.12) sleep stories per week, which corresponds to 60.33 (SD 5.75) minutes of meditation, 8.41 (SD 11.23) minutes of music, and 4.63 (SD 5.36) minutes of sleep stories. Table 4 shows the breakdown of weekly use of specific types of meditation. Across all weeks, series meditations were the most used content type.

Finally, as shown in Tables 5 and 6, linear regression models demonstrated that the average weekly app use (measured both as average minutes per week and average sessions per week) was not significantly associated with poststudy reports of enjoyment using the app, ease of app use, satisfaction with app content, or satisfaction with the app’s aesthetics and graphics.

**Table 1:** Participant demographic characteristics (N=36).

			Participants, n (%)
**Sex**			
	Female	30 (83)	
	Male	6 (17)	
**Race**			
	White	29 (81)	
	Black or African American	1 (3)	
	Asian or Asian American	3 (8)	
	Alaska Native or Pacific Islander	1 (3)	
	Mixed race	2 (6)	
**Ethnicity**			
	Hispanic or Latino	5 (13)	
	Non-Hispanic or Latino	31 (87)	
**Education**			
	High-school diploma	3 (8)	
	Some college	4 (11)	
	Associate or 2-year degree	3 (8)	
	Bachelor’s degree	13 (36)	
	Graduate school or above	13 (36)	
**Employment**			
	Employed	23 (64)	
	Unemployed or unable to work	9 (25)	
	Retired	2 (6)	
	Homemaker	2 (6)	
**Annual income (US $)**			
	<20,000	2 (6)	
	20,001 to 40,000	1 (3)	
	40,001 to 60,000	5 (14)	
	60,001 to 80,000	7 (19)	
	80,001 to 100,000	2 (6)	
	>100,000	19 (53)	
**Marital status**			
	Single	11 (31)	
	Partnered or in a relationship	3 (8)	
	Married	19 (53)	
	Divorced	3 (8)	
**Type of cancer**			
	Blood	10 (28)	
	Breast	9 (25)	
	Gynecologic	6 (17)	
	Thyroid	4 (11)	
	Kidney	2 (6)	
	Bone	2 (56)	
	Ampullary	1 (3)	
	Appendiceal	1 (3)	
	Rectal	1 (3)	
**First cancer diagnosis**			
	<3 months ago	4 (11)	
	3 to <6 months ago	2 (6)	
	6 months to <1 year ago	7 (19)	
	1 to 3 years ago	18 (50)	
	>3 years ago	5 (14)	

**Table 2:** Average weekly questionnaire responses.

		Study week, n (%)			
		Week 1 (N=22)	Week 2 (N=13)	Week 3 (N=12)	Week 4 (N=11)
**Overall enjoyment with using the meditation app prototype**					
	Very much enjoyed	7 (33)	8 (62)	7 (58)	6 (55)
	Enjoyed	7 (33)	3 (23)	3 (25)	3 (27)
	Neutral	4 (19)	1 (8)	2 (17)	1 (9)
	Did not enjoy	1 (5)	1 (8)	0 (0)	0 (0)
	Did not enjoy at all	2 (10)	0 (0)	0 (0)	0 (0)
	Did not respond	0 (0)	0 (0)	0 (0)	1 (9)
**Overall ease of use of the meditation app prototype**					
	Very easy to use	9 (43)	9 (64)	5 (42)	6 (55)
	Easy to use	6 (29)	1 (7)	5 (42)	2 (18)
	Neutral	3 (14)	2 (14)	1 (8)	2 (18)
	Difficult to use	1 (5)	2 (14)	0 (0)	1 (9)
	Very difficult to use	2 (10)	0 (0)	0 (0)	0 (0)
**How satisfied were you this week with the meditation content?**					
	Very satisfied	10 (45)	7 (50)	3 (25)	5 (45)
	Satisfied	9 (41)	6 (43)	8 (67)	6 (55)
	Neutral	2 (9)	1 (7)	1 (8)	0 (0)
	Dissatisfied	1 (5)	0 (0)	0 (0)	0 (0)
	Very dissatisfied	0 (0)	0 (0)	0 (0)	0 (0)
**How satisfied were you this week with the sleep content?**					
	Very satisfied	3 (38)	3 (50)	1 (14)	2 (40)
	Satisfied	3 (38)	1 (17)	5 (71)	2 (40)
	Neutral	2 (25)	1 (17)	1 (14)	1 (20)
	Dissatisfied	0 (0)	1 (17)	0 (0)	0 (0)
	Very dissatisfied	0 (0)	0 (0)	0 (0)	0 (0)
**How satisfied were you this week with the music content?**					
	Very satisfied	3 (30)	3 (33)	1 (20)	1 (17)
	Satisfied	6 (60)	5 (56)	3 (60)	4 (67)
	Neutral	1 (10)	1 (11)	0 (0)	1 (17)
	Dissatisfied	0 (0)	0 (0)	1 (20)	0 (0)
	Very dissatisfied	0 (0)	0 (0)	0 (0)	0 (0)
**How satisfied were you this week with the breathing exercises content?**					
	Very satisfied	4 (27)	4 (40)	1 (25)	2 (40)
	Satisfied	8 (53)	5 (50)	3 (75)	3 (60)
	Neutral	3 (20)	1 (10)	0 (0)	0 (0)
	Dissatisfied	0 (0)	0 (0)	0 (0)	0 (0)
	Very dissatisfied	0 (0)	0 (0)	0 (0)	0 (0)

**Table 3:** Poststudy questionnaire responses (N=11).

			Participants, n (%)
**Overall enjoyment with using the meditation app prototype**			
	Very much enjoyed	6 (55)	
	Enjoyed	4 (36)	
	Neutral	1 (9)	
	Did not enjoy	0 (0)	
	Did not enjoy at all	0 (0)	
**Overall ease of use of the meditation app prototype**			
	Very easy to use	7 (64)	
	Easy to use	3 (27)	
	Neutral	0 (0)	
	Difficult to use	1 (9)	
	Very difficult to use	0 (0)	
**Overall satisfaction with meditation app content**			
	Very satisfied	6 (55)	
	Satisfied	4 (36)	
	Neutral	1 (9)	
	Dissatisfied	0 (0)	
	Very dissatisfied	0 (0)	
**Overall satisfaction with meditation app aesthetics and graphics**			
	Very satisfied	7 (64)	
	Satisfied	2 (18)	
	Neutral	0 (0)	
	Dissatisfied	1 (9)	
	Very dissatisfied	1 (9)	
**Overall, how helpful was using the meditation app prototype in improving or managing symptoms or difficulties related to your cancer?**			
	Very helpful	4 (36)	
	Somewhat helpful	6 (55)	
	Not too helpful	1 (9)	
	Really not helpful	0 (0)	
**I thought that the length of the meditation sessions was...**			
	Just right	9 (82)	
	Too long	0 (0)	
	Too short	2 (18)	
**Did you use the reminder feature when using the meditation app prototype?**			
	Yes	7 (64)	
	No	4 (36)	
**How useful were the reminders to you?**			
	Very useful	4 (36)	
	Somewhat useful	3 (27)	
	Really not very useful	0 (0)	
	Not applicable	4 (36)	
**Did you use the tracking-streaks feature offered in the meditation app prototype?**			
	Yes	7 (64)	
	No	4 (36)	
**How useful did you find the tracking streaks feature in promoting regular meditation or use of the app?**			
	Very useful	4 (36)	
	Somewhat useful	3 (27)	
	Really not very useful	0 (0)	
	Not applicable	4 (36)	
**Did you use the share status feature offered in the meditation app prototype?**			
	Yes	10 (91)	
	No	1 (9)	
**If yes, how often did you share your status with friends or on social media?**			
	Very often	0 (0)	
	Often	0 (0)	
	Occasionally	1 (9)	
	Never	0 (0)	
	Not applicable	10 (91)	
**Would you recommend using the meditation app prototype for other patients with and survivors of cancer?**			
	Yes	13 (100)	
	No	0 (0)	
**How likely are you to continue using the meditation app prototype in the future?**			
	Extremely likely	7 (64)	
	Likely	4 (36)	
	Unlikely	0 (0)	
	Extremely unlikely	0 (0)	
**Please rate your satisfaction with the *Calm for Cancer* Facebook group**			
	Very satisfied	0 (0)	
	Satisfied	2 (22)	
	Neutral	7 (77)	
	Dissatisfied	0 (0)	
	Very dissatisfied	0 (0)	

**Table 4:** App use by study week and meditation type.

		Study week			
		Week 1	Week 2	Week 3	Week 4
**Completed sessions, mean (SD)**					
	Master class	0.11 (0.58)	0.30 (1.34)	0.00 (0.00)	0.00 (0.00)
	Series	6.15 (5.48)	4.05 (3.73)	3.12 (4.06)	3.46 (4.16)
	Freeform	0.04 (0.19)	0.00 (0.00)	0.12 (0.49)	0.15 (0.55)
	Sequential	0.04 (0.19)	0.45 (1.61)	0.12 (0.49)	0.38 (1.39)
**Total minutes used, mean (SD)**					
	Master class	2.28 (11.87)	4.68 (20.94)	0.00 (0.00)	0.00 (0.00)
	Series	63.09 (67.44)	48.34 (58.77)	49.79 (88.90)	51.45 (78.37)
	Freeform	0.33 (1.70)	0.00 (0.00)	1.47 (6.06)	3.19 (11.51)
	Sequential	0.48 (2.50)	6.35 (22.99)	1.53 (6.31)	8.35 (30.09)

**Table 5:** Linear regression models exploring associations between minutes of app use and participants’ overall enjoyment, ease of use, satisfaction with content, and satisfaction with aesthetics and graphics.

Feasibility outcome	b (SE; 95% CI)	*t* test (*df*)	*P* value
Enjoyment with using the app	−0.001 (0.001; −0.003 to 0.001)	−0.87 (12)	.40
Ease of use of the app	−0.002 (0.001; −0.005 to 0.001)	−1.75 (12)	.11
Satisfaction with app content	−0.001 (0.001; −0.003 to 0.001)	−0.90 (12)	.38
Satisfaction with aesthetics	−0.002 (0.002; −0.006 to 0.001)	−1.34 (12)	.20

**Table 6:** Linear regression models exploring associations between total completed app sessions and participants’ overall enjoyment, ease of use, satisfaction with content, and satisfaction with aesthetics and graphics.

Feasibility outcome	b (SE; 95% CI)	*t* test (*df*)	*P* value
Enjoyment with using the app	−0.016 (0.026; −0.073 to 0.040)	−0.63 (12)	.54
Ease of use of the app	−0.043 (0.030; −0.109 to 0.022)	−1.45 (12)	.17
Satisfaction with app content	−0.020 (0.026; −0.076 to 0.035)	−0.79 (12)	.44
Satisfaction with aesthetics	−0.039 (0.042; −0.131 to 0.052)	−0.93 (12)	.37

### Qualitative Themes From Interviews

Because the qualitative research was based on a small subsample of participants, many of the responses were quite specific to individuals, and the findings are therefore reported in some detail within themes, along with the number of participants providing different types of responses. Verbatim quotes were also used to illustrate key points and capture the experiences of the participants in their own words.

### Overall Experience of Using the App

Of the 6 interview participants, 4 (67%) reported very positive experiences of using the app:

I actually loved it. I loved everything about it. It was way more than I thought what meditation would be about. Like I said, it opened my eyes to the new world of meditation. I just thought it was fabulous.

A total of 33% (2/6) of participants indicated that their experiences had been more mixed, with some aspects that they had not enjoyed. One of the participants with mixed experiences explained that they preferred to use faith-based practices to cope with the experience of having cancer. Because the non–faith-based app did not resonate so much with this participant, meditating for the study felt “more like homework,” although they did report some benefits, “I think in some ways it helped, it was relaxing as a whole, but it’s not my usual go-to.*”* The other participant who reported mixed experiences explained that these were due to the features of the app that they had disliked, such as some of the narrators’ voices or difficulties in navigating the app. Regardless of their overall level of satisfaction with the app, all 100% (6/6) of participants were able to identify features that they disliked as well as those that they liked, as reported in the following sections.

### What Participants Liked About the Calm for Cancer App

#### Specific Narrators

When asked what they had particularly liked about the *Calm for Cancer* app, 67% (4/6) of interviewees mentioned the specific narrators (ie, Tamara Levitt and Teri Richardson) that they had especially enjoyed listening to because of their voices, content, or relatability.

In all, 50% (3/6) of interviewees stressed the importance of relatability when selecting narrators for an app designed for patients with cancer:

I think that that goes a long way...to the patients because they like to know, “Oh, wow, somebody understands, has been through the same thing,” maybe not in the same way, but it just makes someone feel a little bit better about—whether it’s using the app or telling someone their story. There’s a relationship there.

#### Types of Content

Of the 5 participants who mentioned that they had particularly liked certain types of meditations, 3 (60%) said that they liked the guided meditations, with one stressing the value of having guided meditations available that were tailored to their mood:

I did appreciate that there were guided meditations depending upon the emotions you were feeling or going through.

Other types of content that were particularly liked by the participants were those that taught them either how to meditate or about other practices that helped them cope with the experience of having cancer, such as gratitude:

It went through steps on gratitude and how it, being grateful meant in the long run would help you throughout your cancer...That was my number one that I loved.

Individual participants also mentioned the meditation series and storytelling as features of the app that they liked.

#### Functions and Features

In all, 67% (4/6) of interviewees mentioned one or more specific features of the app they liked, with 6 different features mentioned among them, and 33% (2/6) of participants mentioned that they liked the visual appearance of the app, which was clear and simple to navigate. Other liked features mentioned by individual participants were mood check-in, automatic reminder notifications, the option to rate the meditations, the use tracker, and automatic bookmarking that suggested the next meditation in a series:

I do like that it wasn’t very overwhelming. It was just straight forward and accessible.

I thought it was very user friendly, that I was able to navigate around in it pretty easily.

#### Sound, Music, and Voices

In all, 67% (4/6) of interviewees mentioned that they liked the app’s background music, sound effects, or narrators’ voices, finding them soothing and relaxing:

I found the voice to be...just perfect. It wasn’t irritating. It was very calm. He was very calm.

#### Variety of Meditation Options

Finally, 33% (2/6) of participants highlighted that they liked having such a wide range of options to choose from when meditating, both in terms of the length of meditation (1 participant) and the variety of topics (2 participants):

I liked that there were many options. I was clicking around...If I didn't feel like I was getting into something, then I could click into something else and then focus.

Table 7 shows the number of participants who gave particular types of responses when asked what they liked about the Calm for Cancer app.

**Table 7:** Features liked by the participants.

Theme and subtheme			Participants, n (%)
**Favorite narrators (N=6)**			
	Favorite narrators	4 (67)	
	Relatability	3 (50)	
**Types of content (N=5)**			
	Guided meditations	3 (60)	
	Educational or topic-based	1 (20)	
	Series	1 (20)	
	Story-based	1 (20)	
**Specific functions and features (N=4)**			
	Visual appearance	2 (50)	
	Rating of meditations	1 (25)	
	Automatic bookmarking	1 (25)	
	Mood check-in	1 (25)	
	Reminder notifications	1 (25)	
	Use tracker	1 (25)	
**Sounds, music, or voices (N=4)**			
	Music	2 (50)	
	Narrator voices	2 (50)	
	Background sleep sounds	1 (25)	
**Variety of meditation options (N=2)**			
	Range of topics	2 (100)	
	Different lengths	1 (50)	

### What Participants Disliked About the Calm for Cancer App

The study participants were also asked if there was anything that they had disliked about the *Calm for Cancer* app or what their least favorite features were. In all, 83% (5/6) of participants mentioned specific features or content in response to this question, although the remaining participant (1/6, 17%) could not recall anything they disliked about the app.

#### Narrator Voices or Styles

Of the 5 participants who disliked some aspects of the app, 4 (80%) mentioned that they disliked the voices or narrative styles of some of the narrators and found these irritating or nonconducive to a relaxed meditative state. A participant mentioned that although they had enjoyed the bedtime story meditations, they were challenging and difficult to follow:

...I think it would be better to have that more soothing voice, that less talking, less interaction and just try to focus on what is it that I'm trying to focus.

The voices to me were not relaxing...they just didn't work for me.

#### Other Features or Content

A total of 4 other aspects of the app were mentioned by individual participants when asked what they had disliked or what their least favorite features were. A participant referred to mood check-in, which they initially liked but had become irritated, as the purpose of this was unclear:

I got tired of doing the check-ins because I had the same little smiley face every time. I didn’t really understand...I didn’t really connect that, so that was probably my least favorite.

For another participant, having so many categories and meditations to choose from had been a little overwhelming and their least favorite aspect of the app. This participant also mentioned that they had disliked a meditation series in particular:

Too many categories, too many choices. That was a little bit hard to go through and try to pick and choose which ones best.

Finally, a participant referred to the tracker function, which sometimes increased their anxiety:

I don’t like to see myself fail, so if I saw I missed a day or something...It would actually cause more anxiety for me.

Table 8 shows the number of participants who mentioned particular factors when asked what they liked the least about the Calm for Cancer app.

**Table 8:** Features disliked by the participants.

Theme and subtheme		Participants, n (%)
Specific narrator voices or styles (N=4)		4 (100)
**Other features or content (N=3)**		
	Mood check-in	1 (33)
	Use tracker	1 (33)
	Too many choices	1 (33)
	Specific meditation series	1 (33)

### Ease of Access and Use

The interviews explored the participants’ views on ease of use of the app and how easy they felt it would be for other patients with or survivors of cancer to access and use it. All 100% (6/6) of participants reported that they found the app easy to access and use. A participant mentioned that they liked the simplicity of the app, which could be used at any time or place without any special arrangements:

I don’t mind going into a quiet space and participating, but to actually have a place in a house where I set up and have this own mediation corner is more difficult. I don’t think you need to do that necessarily to participate in the app.

Only 33% (2/6) mentioned that they had experienced any difficulties in navigating content on the app, although these individuals also stressed that, in general, they had found the app easy to use. All 100% (6/6) of participants also indicated that they felt that other patients with or survivors of cancer would have no difficulty in accessing or using the app, although a participant stressed that older people with little experience in using apps might have a little more difficulty:

Depending on the age, younger ones no problem whatsoever. You start getting into people that are maybe Baby Boomer age...it’s probably a little bit more for them to learn. Anybody who’s familiar with apps, it’s pretty easy.

### Mental or Physical Benefits

In all, 83% (5/6) of participants mentioned that using the app helped them deal with the stress or anxiety of their condition:

Before my diagnosis, I had anxiety. That was something that was helpful for me to have this schedule at night to listen to these meditations and get out of my own thoughts for a minute and just really focus.

I felt myself not only calm during the app, during a meditation, but I felt myself more calm throughout the day.

A total of 33% (2/6) of participants had experienced better sleep when using the app to meditate:

I was waking up feeling better because I was going to sleep better, instead of reading and being on my phone for an hour and then just rolling over and trying to go to sleep. I think having this schedule and listening to this before bed helped with the overall night of sleep, for sure.

When asked specifically whether the app had helped them to sleep better, 50% (3/6) of participants reported that they had not tried using it for this purpose, with 67% (2/3) of these explaining that coexisting health conditions or their cancer treatments have a negative impact on sleep, which they did not think meditation could overcome. In addition, 33% (2/6) of participants expressed the view that their religious beliefs or existing lifestyle practices helped them cope with the experience of having cancer and that using the app did not provide any clear additional benefits. None of the participants reported any ways in which using the app helped them deal with physical pain, although several stressed that their cancer did not cause any pain.

### Ideal Length of Meditations

When asked for their views on the ideal length of meditation for patients with cancer, the responses ranged from 5 to 15 minutes. Most participants (4/6, 67%) expressed the view that approximately 10 to 15 minutes is the best length.

In addition, 33% (2/6) of participants indicated that they felt shorter durations for meditation (ie, 5-8 minutes) would be more appealing to users or easier to fit into a busy schedule, used either once or more than once daily.

### Suggested Improvements

When asked for suggested improvements to the *Calm for Cancer* app, 83% (5/6) of participants provided 22 separate suggestions, which were categorized as relating to navigation (2 participants), content (4 participants) or style (2 participants). Suggestions for navigation included the ability to save or bookmark favorite meditations, improved search function or visibility, different categories of meditations for different users, and the length of meditations shown. Suggestions for the content included religion, metastatic disease, and depression. Suggested improvements for style included the choice of surroundings or backgrounds for meditations and longer meditations (approximately 15 minutes):

I think it might be helpful to break out the category for the different populations. For example, cancer patient versus caretakers, but also...my condition is not as severe as others. I think that if there was a different category for different people that have different daily experiences, I think would be helpful.

I didn’t see in the app where it covered depression...that would be something I would think to zero in on, and it would help people with cancer possibly deal with it more ’cause there’s a lot of depression with...just the medications you’re on.

### Intentions to Continue Meditation

When asked whether they intended to continue their meditation practice in future, 67% (4/6) of participants indicated that they definitely did, with 33% (2/6) explaining that they had already purchased the main *Calm* app or another app for use in their practice. However, several of those who expressed an intention to continue meditating admitted that they had not been very successful in adhering to a regular meditation schedule since the conclusion of the study:

I downloaded the overall Calm app. I haven't been as successful as I'd like in using it.

A participant expressed uncertainty about whether they would continue with the app, whereas another indicated that they did not intend to continue mediating.

### Wider Market for the App

All but one of the participants indicated that they would download the *Calm for Cancer* app if it became publicly available. In all, 50% (3/6) of participants expressed the view that the app would be of considerable interest to other patients with or survivors of cancer and should be made more widely available:

I just feel like somebody always knows somebody that’s going through cancer or can recommend it. Yeah, I think it would catch on. I think people would pick it up.

## Discussion

### Principal Findings

We assessed the feasibility of a cancer-specific meditation mobile app prototype, *Calm for Cancer*, among patients with and survivors of cancer, using questionnaires, objective use data, and in-depth interviews. Both quantitative and qualitative data demonstrated the overall feasibility of the *Calm for Cancer* app in patients with and survivors of cancer. Weekly and poststudy questionnaire data showed that users reported high overall enjoyment, ease of use, and satisfaction with the app content, aesthetics, and graphics. The emergent top-level themes arising from the poststudy interviews included the overall experience of using the app, what participants liked about the app, what participants disliked about the app, ease of access or use, mental or physical benefits, ideal length of meditations, suggested improvements, intentions to continue meditation, and wider market for the app. The objective use data further showed that, on average, participants used the app for 73 (SD 7.1) minutes per week, demonstrating compliance with use prescriptions (ie, 70 minutes per week). Overall, this feasibility RCT provides insight into considerations for the final app design and serves as an important step before testing the efficacy of the *Calm for Cancer* meditation app.

Overall, both weekly and poststudy questionnaire responses indicated high feasibility across the following categories: (1) acceptability (ie, satisfaction, perceived appropriateness, and perceived positive and negative effects), (2) demand (ie, use of the app, interest, or intention to use), and (3) practicality (ie, how it makes them feel and ease of use). In addition to the self-reported survey data, objective participation data also supported the demand, as participants were compliant with the prescription app use of 70 minutes per week. Previously, no commercially available meditation apps have been specifically developed for the needs of patients with cancer and designed with input from end users at both the individual and clinic levels (ie, patients with and survivors of cancer and health care providers), making the *Calm for Cancer* prototype the first of its kind. In 2019, a feasibility RCT of the parent *Calm* app was conducted among patients with hematologic cancer, who were asked to use the *Calm* app for 10 minutes per day for 4 weeks [6]. The study found that the use of the *Calm* app was feasible among patients with hematologic cancer; specifically, 83% of participants reported enjoyment, 84% reported being satisfied with the content, and 97% reported that they would recommend it to others, and the average objective app use was 71 (SD 74) minutes per week [6]. However, despite general satisfaction, poststudy qualitative interviews revealed strong recommendations for adapting the app more specifically for cancer [5]. Compared with our 2019 feasibility RCT assessing the feasibility of the general *Calm* app that did not include cancer-specific content or modifications to meet the needs of patients with cancer, participants using the *Calm for Cancer* app reported greater satisfaction with the prototype app after 4 weeks (eg, higher enjoyment, higher satisfaction with content, and greater likelihood of recommending the app to others). In addition, although the objective app use data indicate similar average weekly engagement between this study and the 2019 study [6] (mean 73 minutes per week for the *Calm for Cancer* app vs 71 minutes per week for the general *Calm* app) across the sample and over time, there was much more consistent use of the *Calm for Cancer* app than we previously observed with the general *Calm* app (ie, SD 7 minutes per week for the *Calm for Cancer* app vs SD 74 minutes per week for the general *Calm* app). Furthermore, the previous feasibility RCT of the *Calm* app was limited to hematologic cancer, whereas this study was conducted among a range of diverse cancer types [5]. Overall, our results suggest that the *Calm for Cancer* app is more feasible and may be more suitable than the parent *Calm* app for meeting the needs of patients with and survivors of cancer. This was despite attrition, missing data, and a self-selected study sample.

Our exploratory analyses did not support the hypothesis that greater app use would predict greater satisfaction with the app (ie, enjoyment, ease of use, satisfaction with content, and aesthetics). However, as reported earlier, in addition to the high rates of satisfaction with the app prototype, there was consistently high compliance with use prescriptions across participants and over time (mean 73, SD 7 minutes per week of the prescribed 70 minutes per week). Although these high levels of engagement provide strong support for the feasibility of the app as an intervention for patients with and survivors of cancer, they limit the statistical power of detecting differences in satisfaction based on app use. Despite the lack of power to detect statistically significant differences, these findings show that high levels of app engagement are associated with high satisfaction among end users and clear feasibility of the app as an intervention for patients with and survivors of cancer. Aligned with a substantial body of behavioral research, this study supports the importance of creating effective strategies that help initiate and maintain engagement with behavioral health interventions. For example, previous studies have shown that greater user engagement with a mobile app can lead to greater feasibility outcomes (eg, greater intention to continue using the app, greater likelihood of recommending the app to others, and higher positive ratings of the app) [21]. Given the small sample size in our feasibility RCT, studies that are powered by larger sample sizes are necessary to further explore and establish the potential associations between use and other feasibility outcomes in meditation app interventions among patients with and survivors of cancer.

The qualitative findings from the in-depth interviews with a subsample of participants provided further insight into the overall feasibility of the *Calm for Cancer* app prototype as well as specific feedback related to adaptation (ie, suggestions for modifications to improve the app to better meet the unique needs of patients with and survivors of cancer). Qualitative analyses based on participant interviews (N=6) revealed that most participants had a very positive experience of using the app (n=4, 67%), although some participants expressed mixed responses because they preferred a faith-based approach and disliked the narrators’ voices (n=2, 33%). In addition, the interviews revealed that all participants perceived the app as easy to use (6/6, 100%), most participants found that the app provided mental health benefits (5/6, 83%), and half of the participants thought that the app would be marketable to other patients with and survivors of cancer (3/6, 50%). Specific features of the *Calm for Cancer* app that participants liked included narrators or narrator voices, relatability, guided meditations, educational and topic-based content (especially those related to the emotional and cancer-related challenges they experienced), series, story-based, visual appearance, rating of meditations, automatic bookmarking, mood check-in, reminder notifications, use tracker, music, background sleep sounds, range of topics, and different meditation lengths.

When asked about any dislike, some participants (2/6, 33%) expressed that they disliked the voices or narrative styles of some of the narrators; thus, including more narrators can be explored in the final design of the *Calm* voices or narrative styles for cancer apps so that users have more options for voices and narrative styles. A participant found the bedtime story meditations challenging and difficult to follow, a participant found the purpose of the mood check-in feature unclear, a participant found that the choice of many categories and meditations was overwhelming, and a participant disliked the tracker function; however, most other participants expressed that they liked these features.

Specific recommendations for app adaptations for the planned efficacy trial included adding the ability to save or bookmark favorite meditations, improving the search function or increasing the visibility of the search function, adding different categories of meditations for different users, including content-related metastatic disease and depression, adding options for different surroundings or backgrounds, and adding longer meditations.

The *Calm for Cancer* app prototype capitalized on a partnership with a popular consumer-based app (ie, *Calm*) to contribute to the future success of the cancer-specific meditation app. More specifically, the research team had a long-standing relationship with the *Calm* app, which provided in-kind app subscriptions for the feasibility and pilot RCTs conducted by the research team. This is one of the first prototypes to be developed by leveraging a consumer-based product and is important for several reasons: (1) the *Calm* app has an extremely large reach, name recognition, and a committed user base that will help the *Calm for Cancer* app to be successful in the market [21]. Second, when marketed to cancer care clinics, professionals, and patients with and survivors of cancer, *Calm* offers subscriptions at highly reduced rates. Overall, this partnership allowed the research team to uniquely draw on the strengths of both academic research and the commercial sector by combining rigorous science with industry standards. Leveraging industry partnerships is also efficient and cost-effective in the long term [21], because many researchers do not have access to resources to maintain an app after development or the costs to publish the app to multiple platforms (eg, Apple). Future directions for this line of research include finalizing the *Calm for Cancer* app design and conducting a randomized clinical trial to determine its efficacy and sustainability in reducing symptom burden in patients with and survivors of cancer. After establishing efficacy and sustainability, the app could be marketed to both consumers and clinics working with cancer care providers via our already existing cancer center partnerships.

### Limitations

Despite study strengths, there are some limitations to this research that should be noted. This feasibility study was conducted among a sample that primarily consisted of White women with relatively high socioeconomic status, thereby limiting the generalizability of the study results. Future feasibility RCTs of the *Calm for Cancer* app are warranted in samples such as racial and ethnic minorities, men, and individuals with lower socioeconomic status to assess the feasibility and efficacy of this app specifically among diverse patients with and survivors of cancer. Another limitation of this study was the relatively high attrition rate, given that approximately half of the participants (18/36, 50%) who were enrolled and completed the baseline questionnaire remained in the study through the final poststudy questionnaires. Future research should explore the use of effective retention strategies for participants engaging in mobile health research trials. Finally, a control group was not included, and the sample size was not powered. However, the study design and sample size were appropriate for a feasibility RCT.

### Conclusions

This study established the feasibility of *Calm for Cancer*, a cancer-specific mobile meditation app prototype that was developed with input from patients with cancer, survivors of cancer, and health care providers. Questionnaires and objective use data were used to demonstrate feasibility across the following categories: acceptability, demand, practicality, and adaptation. In addition, qualitative interviews were conducted to gain further insight into the experiences of patients with and survivors of cancer with the meditation app prototype. The options suggested by the participants for additional or revised content, narrators, and length, as well as suggested feature improvements, will be considered in the final app design before testing the *Calm for Cancer* meditation app for its efficacy in a future study.

## Appendix

Multimedia Appendix 1 Poststudy interview questions.

Multimedia Appendix 2 Facebook discussion prompts.

Multimedia Appendix 3 CONSORT-eHEALTH checklist (V 1.6.1).

## Abbreviations

RCT: randomized controlled trial
REDCap: Research Electronic Data Capture
