# Supplementary material for: Meditation Mobile App Developed for Patients With and Survivors of Cancer: Feasibility Randomized Controlled Trial
Source: JMIR Cancer. 2022 Nov 23;8(4):e39228. doi: 10.2196/39228 (PMC9730204; doi:10.2196/39228)
Supplement: Multimedia Appendix 1 [file cancer_v8i4e39228_app1.docx]

**Additional File 1. Post-Study Interview Questions**

1. Tell us about your experience using the meditation app prototype?
2. Please describe whether and how the meditation app helped you (would help patients/survivors) cope with any discomfort or negative health symptoms?
   1. Fatigue?
   2. Mental health (e.g., stress, anxiety, depression)?
   3. Sleep?
   4. Pain?
   5. Other behaviors?
3. What was your favorite part about the app?
4. What was your least favorite part about the app?
5. Were there things that were missing from your experience with the app that you would have benefitted from?
6. Did you find the meditation app prototype easy to use? Describe why or why not.
7. What do you think is the ideal amount of time to use the app?
   1. Minutes per session? Days per week?
8. Did you have a favorite or least favorite meditation? Why?
   1. Favorite/least favorite teacher? Why?
9. Did you find the meditation guidance helpful and enticing for continuing a meditation practice?
10. Is there anything that should be changed to make the app helpful and enticing for continuing a meditation practice?
    1. If this app was available now, do you think you would download it? Why?
11. Any future plans for meditation practice?
12. Do you think other cancer patients/survivors will have any difficulty accessing or using the meditation app? Describe why or why not.
13. What changes would you make to the meditation app?
