# Supplementary material for: Meditation Mobile App Developed for Patients With and Survivors of Cancer: Feasibility Randomized Controlled Trial
Source: JMIR Cancer. 2022 Nov 23;8(4):e39228. doi: 10.2196/39228 (PMC9730204; doi:10.2196/39228)
Supplement: Multimedia Appendix 2 [file cancer_v8i4e39228_app2.docx]

**Additional File 2. Facebook Discussion Prompts**

1. How are you feeling about your meditation practice overall?
2. What are some barriers you are currently experiencing during your meditation practice? How are you overcoming them?
3. What are you enjoying most about meditating with Calm for Cancer?
4. What is your favorite and least favorite part of the meditation app prototype thus far?
5. What did you think about the meditations you did this week? Were they challenging in any way?
6. Are there any meditations that you enjoyed so much you would want others who have cancer or survived cancer to listen to? Which ones and why?
7. When do you find it easiest to incorporate meditation into your schedule? How is it easy?
8. Do you feel like you are getting more comfortable meditating? What has been the hardest part? The easiest?
9. How do you think meditation may affect, or has affected, you thus far?
10. Some individuals find a certain meditation class or session they “love” and others they try to avoid. Have any specific meditation on the app really stuck with you and make you want to do it over and over again?
11. What was one “win” you experienced with your meditation practice this week?
12. What is some advice you would give someone with cancer who wanted to start meditating?
13. What aspects of the Calm for Cancer app (e.g., Breath Bubble, Sleep Stories, Meditations, Music) do you like most and why?
14. Do you utilize any of the check-in features (mood, gratitude, sleep)? Which ones are helpful and why?
15. Did you ever use the reminders feature to help you remember to meditate or use the check-in features? Were these helpful? Why? Can you think of other strategies that could be integrated into the app to help you meditate more?
16. What is the best feature of the meditation app prototype?
17. What is the worst or least useful feature of the meditation app prototype?
